# Expression of human leukocyte antigen-G and acute rejection in patients following liver transplantation

**DOI:** 10.3892/etm.2014.1917

**Published:** 2014-08-19

**Authors:** WEI-YU HU, LI-QUN WU, ZHAN SU, XU-FENG PANG, BIN ZHANG

**Affiliations:** Department of Hepatobiliary and Pancreatic Surgery, Affiliated Hospital of Medical College, Qingdao University, Qingdao, Shandong 266003, P.R. China

**Keywords:** liver transplantation, human leukocyte antigen, rejection, acute, diagnosis

## Abstract

Acute liver rejection is one of the most severe complications that may affect the liver transplantation procedure. Thus, one of the most important focal points in the field of liver transplantation research is to discover a non-invasive or less-invasive method of diagnosing and predicting cases of acute liver rejection. In the present study, 59 tissue samples, including blood and liver tissues, were collected from patients who underwent liver transplantation between March 2005 and November 2009. The patients were divided into acute rejection and no rejection groups, the latter of which was further divided into normal and abnormal liver function groups. The samples were assayed by enzyme-linked immunosorbent assay and immunohistochemistry methods. The results were analyzed and a receiver operating characteristic (ROC) curve was plotted. The area under the ROC curve and the sensitivity and specificity of the cut-off point were analyzed statistically. The results indicated that the expression level of human leukocyte antigen-G (HLA-G) in the serum and liver samples in the acute rejection group was markedly lower than that in the no rejection group (P<0.001 and P=0.004, respectively). Furthermore, in the no rejection group, no statistically significant difference was identified in the level of HLA-G between patients with normal or abnormal liver function (P=0.0593). The area under the ROC curve was 0.805. When 2.41 U/ml HLA-G was considered as the cut-off point for the diagnosis of acute liver rejection, the sensitivity and specificity were 72.7 and 83.8%, respectively. In conclusion, in the present study, a high expression of the HLA-G was shown to correlate with a reduced occurrence of acute liver rejection. HLA-G may thus be an effective factor for the diagnosis and prediction of acute liver rejection.

## Introduction

Liver transplantation is a therapeutic strategy for the treatment of patients with end-stage liver disease and it is currently under rapid development (1). However, acute liver rejection following transplantation remains a severe complication of the procedure. Therefore, one of the most important focal points in the field of liver transplantation research is to discover a non-invasive or less-invasive method of diagnosing and predicting cases of acute liver rejection. Human leukocyte antigen-G (HLA-G) is a nonclassical human leukocyte antigen (HLA) class-I molecule that has been revealed to be expressed in placental trophoblast cells (2). Since trophoblasts are at the physical interface between the fetus and its mother, HLA-G may play a role in the provision of maternal immunity to the semi-allogeneic fetus. Despite a growing number of published studies, there remains no consensus on many aspects of HLA-G, including its tissue distribution and receptor binding (2,3). In the current study, the expression of HLA-G in blood and liver tissue samples was analyzed in order to investigate the correlation between the expression of HLA-G and acute rejection in patients undergoing liver transplantation.

## Materials and methods

### Patients

Between March 2005 and November 2009, blood and liver tissue samples were collected from 59 patients during 1–3 months following liver transplantation at the Department of Hepatobiliary Surgery and Pancreatic Surgery of the Affiliated Hospital of Medical College, Qingdao University (Qingdao, China). The present study was approved by the ethics committee of Qingdao University (Qingdao, China). All of the patients have given their consents for this study. The patient cases comprised 51 male and eight female cases aged from 35 to 65 years, with a median age of 45 years. The primary disease in the patients was end-stage liver disease (27 cases), small hepatocellular carcinoma (24 cases) or other liver disease (8 cases). Immunosuppressive treatment consisted of tacrolimus (FK506), methylprednisolone (MP) and mycophenolate mofetil (MMF). The concentration of FK506 was maintained at 10–15 ng/ml for the first three months following liver transplantation. According to their history and physical examinations, liver function, and pathology, the patients were divided into two groups: i) acute rejection group (22 cases, rejection group) where the patients suffered from conditions including fever, restlessness, and debility and displayed increased levels of bilirubin and aminopherase; their pathology was displayed as acute rejection [Banff program, rejection activity index (RAI) ≥4 (3)]; ii) no rejection group (37 cases) where the pathology of the patients revealed no acute rejection (Banff program, RAI ≤2). The patients in the no rejection group were further divided into two groups according their liver function: i) normal liver function group (15 cases, normal group) who had normal levels of bilirubin, aminopherase and albumin; ii) abnormal liver function group (22 cases, abnormal group) who had abnormal levels of bilirubin, aminopherase or albumin.

### Enzyme-linked immunosorbent assay (ELISA)

Serum samples were preserved at −70°C following centrifugation. The level of HLA-G was measured by ELISA in plates coated with the capture antibody MEM-G/09 (HLA-G kit; XiTang Bio-Technology Co., Ltd, Shanghai, China). Following several washes, diluted serum was added to each well (100 μl) in duplicate and the plates were incubated for 1 h. Labeling was performed with the HLA-G kit and the optical densities were measured at 450 nm (Model 550; Bio-Rad, Hercules, CA, USA). The final concentration was determined by optical density according to the standard curves.

### Immunohistochemistry

Liver tissue sections (4 μm thick) were fixed for 10 min in cold acetone, dehydrated and permeabilized with saponin in phosphate-buffered saline (PBS). Endogenous peroxidase blocking was performed using the Dako EnVision system (Beijing Zhongshan Golden Bridge Biotechnology Co., Ltd., Beijing, China), and non-specific binding was performed with 3% low-fat dried milk diluted 1:100 with PBS. Samples were incubated with the primary monoclonal antibody (mAb) anti-HLA-G (4H84 mAb; Santa Cruz Biotechnology, Inc., CA, USA), for 60 min. Subsequently, secondary conjugated goat anti-mouse antibodies (Santa Cruz Biotechnology, Inc.), coupled with peroxidase, were added and incubated for 30 min. Finally, the samples were incubated with 3,3′-diaminobenzidine diluted in 0.01% H_2_O_2_ for 10 min, counterstained with hematoxylin, dehydrated and mounted. To validate the anti-HLA-G mAb and the immunohistochemical method, paraffin-embedded sections of trophoblastic tissue (positive control) were systematically analyzed. The basal expression level of HLA-G was evaluated in normal liver biopsies obtained at autopsy from 10 healthy individuals who had succumbed as a result of violent trauma. A negative control was performed by omitting the primary antibody. The grading criteria for staining intensity were: 1, light yellow; 2, yellow-brown; and 3, brown. The grading criteria for positive percentage were: 0, no positive cells; 1, ≤10%; 2, 21–30%; 3, 31–70%; and 4, >70% positive cells. The accumulated points were calculated as the sum of the above two scores and the grading criteria were: (+), 1–3; (++), 4 or 5; and (+++), 6 or 7.

### Statistical analysis

All statistical analyses were carried out using SPSS software, version 13.0 (SPSS, Inc., Chicago, IL, USA). Data are expressed as medians or the number of patients, and analyzed using t- or χ^2^-tests in the different groups. A receiver operating characteristic (ROC) curve was produced for the acute rejection and no rejection groups.

## Results

### Expression levels of HLA-G in blood samples from different groups

**Figure 1:**
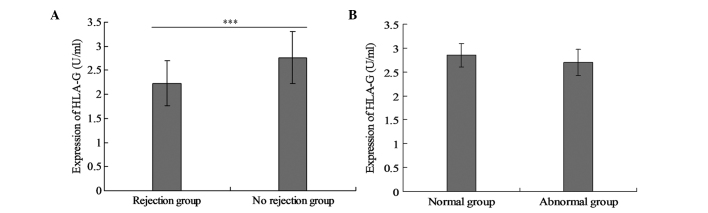
Expression of human leukocyte antigen-G (HLA-G) in blood samples from the different groups. Expression of HLA-G in blood samples from (A) the rejection and no rejection groups and (B) the normal and abnormal groups.

The expression level of HLA-G in the rejection group (2.23±0.47 U/ml) was significantly lower than that in the no rejection group [2.76±0.54 U/ml, P<0.001; Fig. 1]. No statistically significant difference was identified in the HLA-G expression level between the normal (2.85±0.24 U/ml) and abnormal groups (2.70±0.27 U/ml, P=0.084; Fig. 1).

### Levels of HLA-G in liver tissue samples from different groups

**Figure 2:**
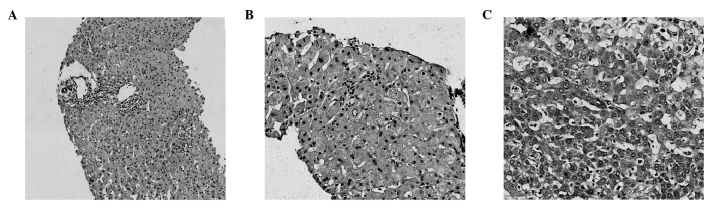
Expression of human leukocyte antigen-G (HLA-G) in the liver tissue. (A) Acute rejection liver tissue, magnification, ×100. (B) Expression of HLA-G in the acute rejection liver tissue (+), magnification, ×200. (C) Expression of HLA-G in the no rejection group liver tissue (++), magnification, ×200.

The expression level of HLA-G in the rejection group was significantly lower than that in the no rejection group (P=0.004). No statistically significant difference was identified between the normal and abnormal groups (P=0.0593). The expression levels of HLA-G in the blood samples had comparable trends to those in liver tissues (Table I and Fig. 2).

### Cases of liver transplantation negatively correlated with the expression level of HLA-G in the rejection group

**Figure 3:**
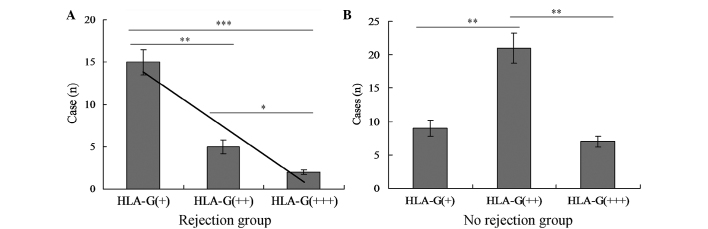
Correlation between number of cases of liver transplantation and human leukocyte antigen-G (HLA-G) expression. (A) Correlation in the rejection group. (B) Correlation in the no rejection group. ^*^P<0.05, for a comparison between the HLA-G(++) and HLA-G(+++) groups; ^**^P<0.01, for a comparison between the HLA-G(+) and HLA-G(++) groups; ^***^P<0.001, for a comparison between the HLA-G(+) and HLA-G(+++) groups.

Fig. 3 shows that the majority of the liver transplantation cases in the rejection group were classified as HLA-G(+) and that the lowest incidence of liver transplantation cases with rejection was in the HLA-G(+++) group. Notably, there was a linear correlation between the number of cases and the expression level of HLA-G (Fig. 3). However, there was no correlation between the number of liver transplantation cases and the expression level of HLA-G in the no rejection group.

### ROC curve of HLA-G in blood

**Figure 4:**
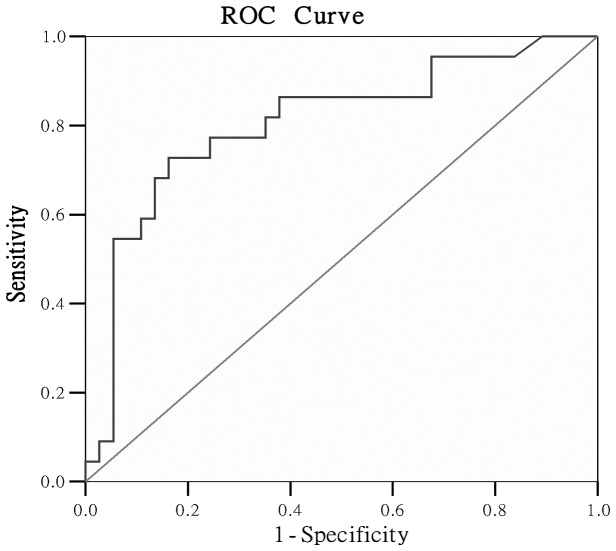
Receiver operating characteristic (ROC) curve of human leukocyte antigen-G (HLA-G) in the rejection and no rejection groups.

In the present study there were 22 cases in the acute rejection and 37 cases in the no rejection groups. Using SPSS software, an ROC curve (Fig. 4) was plotted comparing the expression level of HLA-G in patients in the acute rejection group with that in the no rejection group. The area under the curve was 0.805 (95% confidence interval = 0.682–0.927). The point on the ROC curve with the maximal Youden Index (YI; YI = sensitivity + specificity −1) (4) was defined as the optimal cut-off point to diagnose rejection. A HLA-G concentration of 2.41 U/ml was selected as the cut-off point from the table produced by the SPSS software. Acute liver rejection was considered when the expression level of HLA-G in the patients’ blood was lower than the cut-off point. The sensitivity and specificity were 72.7 and 83.8%, respectively.

## Discussion

HLA-G is a nonclassical HLA class-I molecule that is selectively expressed on cytotrophoblast cells at the maternal-fetal interface. HLA-G was first discovered as a ligand for the inhibitory receptors present on uterine natural killer (NK) cells, thereby contributing to maternal-fetal tolerance. HLA-G differs from other HLA class-I molecules by its: i) lower polymorphism; ii) restricted tissue distribution; iii) particular expression pattern due to the seven protein isoforms generated from alternative splicing, and iv) biological properties that lead to immune tolerance (5).

Numerous studies have demonstrated that the expression of HLA-G is higher in patients with immune tolerance following transplantation than in those with decreased immune tolerance (2). A study by Onichtchouk *et al* (6), which explored the association between HLA-G and immune tolerance, reported that HLA-G was observed in 16% (5 cases) of cardiac muscle tissues taken from 31 patients undergoing heart transplantation. There was less acute rejection in patients who were positive for HLA-G than in patients who tested negative for HLA-G. Furthermore, no chronic rejection took place in patients positive for HLA-G. Thus, the study concluded that the high expression of HLA-G in patients following transplantation resulted in improved immune tolerance. However, to the best of our knowledge, an association between the expression of HLA-G and liver function has not previously been reported.

In the present study, HLA-G levels were observed in the peripheral blood and liver tissues. There were fewer acute rejections in the group with a high expression level of HLA-G and the level of HLA-G was not found to correlate with liver function. The results revealed that HLA-G would be present regardless of whether the organ was accepted or rejected by the patient. Certain other studies support these results (7–9). Soluble HLA-G (sHLA-G) induces the apoptosis of antigen-specific T lymphocytes via p56lck, calcium calmodulin kinase II and calcium-independent protein kinase C signaling pathways as well as the nuclear translocation of nuclear factor κ-light-chain-enhancer of activated B cells (NF-κB) and nuclear factor of activated T-cells (NFAT) (10). HLA-G activates NF-κB in NK cells (11). Human leukocyte antigen-G5 (HLA-G5) inhibits the cell cycle progression of alloreactive T cells by decreasing the expression of cyclins and upregulating the expression of the cyclin-dependent kinase inhibitor, p27kip (12). HLA-G expression by target cells prevents the polarization of cytolytic granules at the NK-cell immunological synapse (13). HLA-G controls the maturation and migration of dendritic cells (DCs) via immunoglobulin-like transcripts (ILTs) by downregulating the antigen presentation to native T cells and the expression of chemokine receptors that control the trafficking of DCs (14). HLA-G induces T regulatory (Treg) cells through two distinct processes: by the differentiation of native T cells into cluster of differentiation (CD)3 + CD4 low and CD3+ cluster of differentiation (CD8) low suppressor T cells (15,16), or by the rapid transfer of HLA-G from antigen-presenting cells (APCs) to T cells, converting them into temporary HLA-G-positive suppressor cells (17). Furthermore, a novel population of naturally occurring HLA-G-positive Treg cells, that constitutively express HLA-G, exist as a discrete peripheral blood subset in healthy donors and appear to emerge from the thymus (18). HLA-G induces suppressive NK cells through trogocytic acquisition of HLA-G from tumor to NK cells (19).

The ROC curve is considered to be effective for describing and comparing the accuracy of a study. A study by Swets (20) suggested that an area of <0.5 under the ROC curve indicates that an experiment had no diagnostic value; ~0.5–0.7 indicates low reliability; ~0.7–0.9 indicates reliability and >0.9 indicates good reliability. In the present study, the area under the ROC curve was 0.805, suggesting that the experiment was reliable. The results obtained revealed that the area under the ROC curve was a good index for the diagnosis or prediction of acute liver rejection, and a cut-off point of 2.41 U/ml HLA-G was identified as the borderline. Acute liver rejection was diagnosed when a lower level than the cut-off point was measured. However, the results obtained in the present study require confirmation by further studies due to the low sample size used in the current study.

## Figures and Tables

**Table I:** Levels of HLA-G in liver tissues from the different groups.

		HLA-G				
				
Group	Number	+	++	+++	χ^2^ value	P-value
Rejection group	22	15	5	2	11.023	0.004
No rejection group	37	9	21	7		
Normal group	15	3	8	4	1.046	0.0593
Abnormal group	22	6	13	3		

HLA-G, human leukocyte antigen-G.
